# Smoking tobacco prevalence among college students in the Kingdom of Saudi Arabia: Systematic review and meta-analysis

**DOI:** 10.18332/tid/105843

**Published:** 2019-04-19

**Authors:** Saad A. Alotaibi, Mohammed A. Alsuliman, Praveen K. Durgampudi

**Affiliations:** 1 Old Dominion University, Norfolk, United States

**Keywords:** tobacco smoking, prevalence, college campuses, KSA, metaanalysis

## Abstract

**INTRODUCTION:**

During the last two decades, several studies have been published regarding the prevalence of tobacco smoking among college students in the Kingdom of Saudi Arabia (KSA). This systematic review and meta-analysis is intended to determine and examine the smoking prevalence in Saudi college students from 2010–2018.

**METHODS:**

PubMed, Science Direct, APA PsycNET, Web of Science, and CINAHL were used to search for published articles reporting the smoking prevalence among Saudi college students. After eliminating irrelevant articles, investigators independently assessed the quality of each article, based on Russell & Gregory guidelines. MetaXL software was used to calculate the pooled prevalence among included studies, using the IVhert model. Heterogeneity among the included studies was evaluated, using I^2^ statistic. Sensitivity analyses were conducted between male and female genders.

**RESULTS:**

Of the 295 published articles, 29 articles used a cross-sectional design to determine smoking prevalence among Saudi college students. Most of the studies were conducted in Riyadh at health-science-related colleges; the rest were performed in different cities and colleges. The meta-analysis showed that the pooled estimate of smoking prevalence among college students in the KSA was 17% (95% CI: 11–23%). Saudi male students had a prevalence rate of 26% (95% CI: 24–29%), whereas for Saudi female students the prevalence was 5% (95% CI: 3–7%).

**CONCLUSIONS:**

Smoking among Saudi college students was higher than in the majority of regional countries (e.g. Iran). Saudi male students had a higher smoking prevalence than Saudi female college students. Additionally, studies that reported a high prevalence targeted students in specific disciplines. Public health authorities in the KSA should develop a surveillance system that monitors the prevalence of tobacco smoking on campuses. A surveillance system of monitoring tobacco use among Saudi college students could be beneficial in determining the degree of the tobacco problem and in improving current tobacco control programs.

## INTRODUCTION

Tobacco use, in its various forms, is responsible for many preventable diseases and deaths1. A 2018 report indicated that tobacco-related diseases killed more than 7 million people worldwide in 2016^1^. It is projected that, if the trend of tobacco consumption persists, 8 million people will die yearly by 2030^2^. Although tobacco use has declined in many developed countries, 80% of the 1.1 billion current smokers who live in low-and-middle-income countries continue to suffer the burden of tobacco-related illness and death^1^. Concurrently, some high-income countries, such as the Kingdom of Saudi Arabia (KSA), were found to have a statistically significant increase in tobacco smoking between 1980 and 2012^3^. The KSA imported more than 3.4 billion US dollars’ worth of tobacco products from 2010 to 2014^4^. Thus, the KSA’s economic burden, due to tobacco consumption, was 20.5 billion US dollars, and 280000 premature deaths occurred from 2001 to 2010^5^.

For the past three decades, the KSA has implemented certain policies to control and reduce tobacco consumption^6,7^. One policy is to ban the use of tobacco products in government and affiliated facilities; these include college campuses, parks, malls, airports and other shared public spaces designated as tobacco-free zones. Another policy imposes 100% taxation on tobacco products. The latest increase in tobacco product prices was implemented in June 2017^6^. In addition to policy-level interventions, non-profit and government-funded tobacco cessation programs have been implemented periodically, across many cities in the KSA to decrease the epidemic of tobacco use by the Saudi population^6^. The Coordinating Committee for Anti-Smoking Associations organized some of these programs to meet its mission of smoking cessation^6^. Above all, the KSA is an Islamic country that considers tobacco smoking a religiously and socially sinful practice. Nevertheless, smoking tobacco among Saudi college students is still a crucial concern^8^.

Although a 2013 Saudi national survey found that the prevalence of smoking across the population was 12.2%^7^, a recent literature review discovered numerous epidemiological studies exploring Saudi college students’ smoking behavior, providing conflicting percentages of smokers versus non-smokers and frequencies of tobacco consumption^8^. The lifestyle associated with college experiences represents a newfound sense of independence for many students, one that makes them more inclined to take part in risky or dangerous behaviors, such as smoking tobacco^9^. For instance, in most high-income countries^10^ those aged 18–24 years (typically, college students) had no substantial change in smoking rate over the past two decades in spite of the decrease in tobacco consumption among both adults and teenagers.

Almutairi^8^ reported studies that examined tobacco smoking behavior among college students in the KSA across diverse locations, diverse genders, and diverse colleges. He found that researchers in the KSA have been unable to come to a consensus about the actual prevalence of smoking among college-age students^8^. As a result, the present systematic review and meta-analysis was intended to critically examine and analyze existing data in order to estimate the pooled prevalence of smoking tobacco among those in higher education in the KSA. The objective was to compare this study’s results to national-level findings for the KSA and to findings from other neighboring countries at the higher education level. The purpose of these comparisons is to understand the overall prevalence of tobacco smoking and its severity within the KSA and within the region. The goal of this study is to inform decision makers, public health researchers and practitioners, and individuals in the communities about the current tobacco problem, so that they can design and ultimately implement effective tobacco control interventions.

## METHODS

This systematic review and meta-analysis is guided by the Preferred Reporting Items for Systematic Review and Meta-Analyses (PRISMA) checklist^11^.

### Definition

In this study, the population and the topic of investigation were restricted to college students who smoked cigarettes, water-pipes (hookahs), and cigars. The researchers excluded other forms of tobacco, such as electronic cigarettes and smokeless tobacco (e.g. snuffing, dipping, and chewing tobacco) because of their irrelevance to smoking behavior or the lack of existing research in their domains. Current smokers were defined as college students who had smoked at least once within the previous 30 days. For the convenience of reporting the findings, study researchers categorized *health-science-related disciplines* as one term, to encompass medicine, dentistry, applied medical sciences, nursing, or pharmacy colleges.

### Search strategy

Two researchers (SA and MA) developed key terms that aligned with the purpose of this study (Supplementary Table 1). These keywords were used to gather literature from five databases: PubMed, Science Direct, APA PsycNET, Web of Science, and CINAHL. Publication years were restricted to include literature published from 2010 to 2018. This time span was selected based upon the findings of a previous literature review, in order to further investigate what has already been contributed in this research domain^8^. No language restriction was used in this study. The literature search in each database was confined to the title, abstract, or both, except for APA PsycNET, where all fields were used. An example of the keywords used for searching PubMed is: (Smoking[Title/Abstract] OR Tobacco[Title/Abstract] OR Cigarette[Title/Abstract] OR Waterpipe[Title/Abstract]) AND (College[Title/Abstract] OR University[Title/Abstract] OR Students[Title/Abstract]) AND (Saudi[Title/Abstract] OR KSA[Title/Abstract]). Data was gathered from 1 February to 1 August 2018. We also sought additional articles that reported the prevalence of smoking among Saudi college students via articles’ references or studies that cited the included articles.

### Selection criteria

The study had three inclusion criteria: 1) a focus on college students in the KSA, 2) data about smoking prevalence, and 3) a score of at least four out of five on the Russell & Gregory^12^ guide. The researchers excluded articles that: 1) pre-dated 2010, 2) were conducted outside of the KSA, 3) used experimental designs, 4) compared tobacco to other addictive substances, 5) focused on smokeless tobacco or electronic cigarettes, 6) scored three points or less, and 7) restricted access to the full text.

### Data extraction

Two researchers (SA and MA) independently conducted an in-depth review of the articles’ titles, abstracts, and full texts. After identifying articles that met all of the inclusion criteria, the researchers met to confirm similar findings. They then independently extracted data (i.e. gender, prevalence, number of smokers, sample size, population of study, and study location) from each article and evaluated them based on exclusion criteria and the Russell & Gregory guidelines^12^. A third investigator (PD) was brought in to resolve disagreements concerning articles’ inclusion, using discussion and critical appraisal.

### Quality assessment

Two researchers (SA and MA) independently rated and assessed the risk of bias and the quality of each article based on the Russell & Gregory guidelines^12^. Articles had to accrue four points out of a possible five in order to be considered in this study. Any article that scored less than four points was excluded after discussion with the third investigator (PD). This exclusion was because the scores of studies with three points or less indicated that they did not maintain some of the fundamental research guidelines: rigor, credibility, trustworthiness, and believability^12^. The Russell & Gregory^12^ five questions are: 1) ‘Was the research question clear and adequately substantiated?’, 2) ‘Was the design appropriate for the research question?’, 3) ‘Was the method of sampling appropriate for the research question and design?’, 4) ‘Were data collected and managed systematically?’, and 5) ‘Were the data analyzed appropriately?’ (Supplementary Table 2).

### Statistical analysis

We reviewed and compiled, using Excel 2016 (Microsoft Corporation, Redmond, CA, USA), the following data: gender, location, population size, number of smokers, name of college, and estimated prevalence of smoking tobacco. In addition, we used MetaXL 5.3 (www.epigear.com) to conduct the meta-analysis that produced graphs. The estimated pooled prevalence of smoking among college students was computed using the model of inverse variance heterogeneity (IVhet) with double arcsine transformation and a 95% confidence interval (CI)^13,14^. Doi et al.^14^ recommended that, unlike random and fixed effects models, the IVhet and double arcsine models should be used to minimize the chance of overestimating the true prevalence and of underestimating the statistical error. The rational explanation for choosing this model was to deal with the issues of variance instability, which could overestimate each study’s weight in the meta-analysis, and to ensure confidence interval boundaries that lay outside the range of 0 to 1^13,14^.

An I^2^ statistic of heterogeneity was used to detect the percentage of variation across studies that resulted from how they were conducted, rather than from natural variation. An I^2^ of 75%, 50%, or 25% indicates that the heterogeneity was high, moderate, or low, respectively^15^. Subgroup analyses were performed, based on gender, to determine any existing differences in smoking prevalence between males and females. We also ran a sensitivity analysis to assess between-study heterogeneity. Outlier studies were excluded, before conducting the meta-analysis based on the Tukey method^16^.

## RESULTS

### Characteristics of the studies

**Figure 1:**
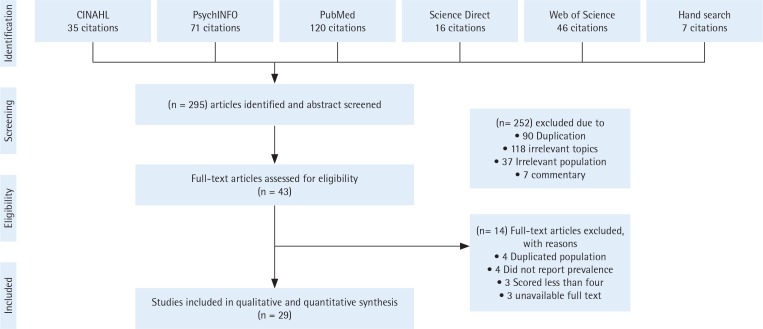
Flow diagram of selection criteria in this systematic review and meta-analysis

Out of the 295 published articles returned by the search method, 29 research articles were included for data synthesis (Figure 1)^17-45^. All of the included studies were cross-sectional descriptive studies that had been carried out primarily within governmental institutions. One study, however, was conducted in a private teaching college^40^. The overall sample size of all included studies was 23237 participants: 12719 males and 10518 females. Riyadh, the capital of the KSA, was the location of 11 (38%) of the studies. Of these 11 studies, 10 were conducted at two universities (King Saud University and King Saud bin Abdulaziz University - Health Sciences).

Of the articles included in this study, 55% examined tobacco smoking prevalence among health-science-related students, while 38% of the articles addressed the smoking prevalence among all college students, including health-science-related majors. The remaining 7% of articles were focused on smoking prevalence within colleges of education (Ed.) and sciences. Among the included studies, 13 (45%) studies measured the prevalence of smoking among both males and females, ten (34%) focused only on males, and six (21%) addressed the prevalence of smoking solely among female students (Table 1).

**Table 1:** Description of all included studies in this systematic review and meta-analysis

*No.*	*Source*	*Gender*	*Prevalence (%)*			*Number of smokers*			*Sample size*			*Population of study*	*Study location*

			*Male*	*Female*	*Total*	*Male*	*Female*	*Total*	*Male*	*Female*	*Total*		
													
1	Abdulghani et al.^17^ (2013)	Female	-	4.3	-	-	39	39	-	907	907	All Colleges	Riyadh
2	Al-Mohaithef & Chandramohan^18^ (2018)	Male	18.7	-	-	63	-	63	337	-	337	All Colleges	Abha
3	Abd El Kader & Al Ghamdi^19^ 2018)	Both	19.2	2.75	9.5	39	8	47	203	291	494	Health Sciences	Jeddah
4	Dar-Odeh et al.^20^ (2017)	Female	-	9.8	-	-	21	21	-	214	214	All Colleges	Al Madinah
5	Azhar & Alsayed^21^ (2012)	Female	-	4.2	-	-	13	13	-	310	310	All Colleges	Jeddah
6	AL-Saegh et al.^22^ (2017)	Female	-	10.3	-	-	32	32	-	310	310	Health Sciences	Jeddah
7	Ansari & Farooqi^23^ (2017)	Female	-	0.9	-	-	3	3	-	332	332	Health Sciences	Dammam
8	Awan et al.^24^ (2016)	Male	23	-	-	123	-	123	535	-	535	Health Sciences	Riyadh
9	Al-Ghaneem & Al-Nefisah^25^ (2016)	Male	30.6	-	-	284	-	284	927	-	927	All Colleges	Majmaah
10	Ansari et al.^26^ (2016)	Male	28.2	-	-	96	-	96	340	-	340	All Colleges	Majmaah
11	Awan^27^ (2016)	Both	-	-	33.8	na*	na*	162	303	177	480	All Colleges	Riyadh
12	Koura et al.^28^ (2011)	Female	-	8.6	-	-	88	88	-	1020	1020	Ed. & Sciences Colleges	Dammam
													
13	Mandil et al.^29^ (2010)	Both	27.5	3.8	14.1	819	141	960	2973	3713	6686	All Colleges	Riyadh
14	Al-Kaabba et al.^30^ (2011)	Both	28.9	4.3	17.6	24	3	27	83	70	153	Health Sciences	Riyadh
15	Allohidan et al.^31^ (2017)	Both	62.5	37.5	24.9	55	33	88	179	175	354	Health Sciences	Riyadh
16	AlQahtani^32^ (2017)	Both	30.1	0.5	30.5	68	1	69	226	207	433	Health Sciences	Najran
17	el-Fetoh et al.^33^ (2016)	Both	88.2	11.8	33.8	90	12	102	160	142	302	Health Sciences	Arar
18	Mansour & Bakhsh^34^ (2015)	Both	39.4	9.4	22.5	56	18	74	142	192	334	Health Sciences	Jeddah
19	Shah & ElHaddad^35^ (2015)	Male	17.3	-	-	66	-	66	380	-	380	All Colleges	Al-Kharj
20	Wali^36^ (2011)	Both	24.8	9.1	14	50	40	90	202	441	643	Health Sciences	Jeddah
21	Mahfouz et al.^37^ (2014)	Both	25.6	4.6	16.8	524	67	591	2165	1599	3764	All Colleges	Jazan
22	AlSwuailem et al.^38^ (2014)	Both	27.8	2.4	17	64	4	68	230	170	400	Health Sciences	Riyadh
23	Al-Haqwi et al.^39^ (2010)	Both	24	0	19	40	0	40	165	50	215	Health Sciences	Riyadh
24	Hassan et al.^40^ (2014)	Male	42.3	-	-	66	-	66	156	-	156	Health Sciences	Riyadh
25	Almogbel et al.^41^ (2016)	Male	24.3	-	-	82	-	82	337	-	337	All Colleges	Buraydah and Hassa
26	Taha et al.^42^ (2010)	Male	15.6	-	-	58	-	58	371	-	371	Health Sciences	Dammam
27	Al-Mohamed & Amin^43^ (2010)	Male	28.1	-	-	388	-	388	1382	-	1382	All Colleges	Hassa
28	Almutairi^44^ (2016)	Male	29.8	-	-	213	-	213	715	-	715	Ed. & Sciences Colleges	Riyadh
29	Torchyan et al.^45^ (2016)	Both	47.6	15.7	32	99	31	130	208	198	406	Health Sciences	Riyadh
	Total					3356	554	4072	12719	10518	23237		
	Pooled estimate (%)		26	5	17								
	95% CI		(24–29)	(3–7)	(11–23)								
	Heterogeneity test (IVhet Model)		I2=78%	I2=90%	I2=97%								
			Q=77.0	Q=154.4	Q=1082.3								
				p<0.001									

na*: not available

### Meta-analysis findings

Among studies that included both males and females, the highest reported prevalence of smoking was 33.8% in two studies^27,33^. Conversely, one study reported the lowest prevalence of 9.5%^19^. Based on gender-specific (i.e. studies surveyed either males or females) studies, 42.3% was the highest prevalence of smoking reported among only male university students^40^, while the lowest prevalence was 15.6%^42^. Among studies reporting only female smoking prevalence, the highest was 10.3%^22^, while the lowest prevalence was 0.9%^23^ (Table 1).

**Figure 2:**
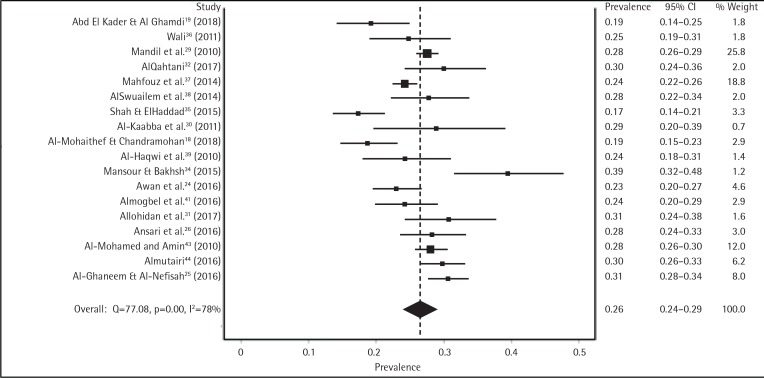
Prevalence of smoking among Saudi male students before conducting sensitivity analysis

**Figure 3:**
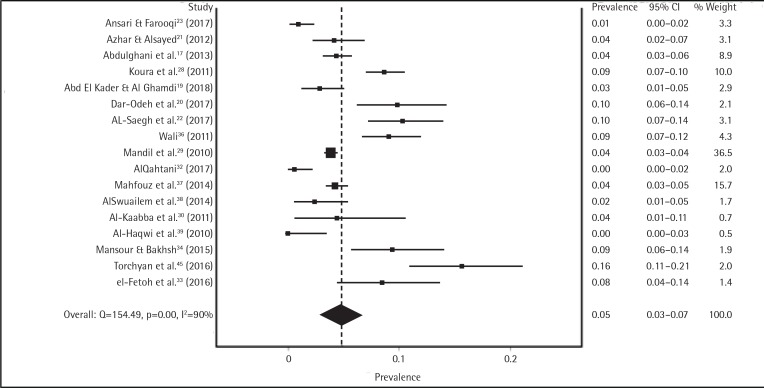
Prevalence of smoking among Saudi female students before conducting sensitivity analysis

After we screened for outlier studies, the meta-analysis revealed that the overall prevalence of tobacco smoking among college students in the KSA was 17% (95% CI: 11–23%) according to the IVhert model. However, the heterogeneity among all included studies was very high (I^2^=97%) (Supplementary Figure 1 and Table 1). Further subgroup analysis was performed, in order to determine the pooled prevalence in each group (male and female) of college students. We found that male and female students had a pooled prevalence rate of tobacco smoking of 26% (95% CI: 24–29%) and 5% (95% CI: 3–7%), respectively. However, the heterogeneity among gender-specific studies remained high: male (I^2^=78%), female (I^2^=90%) (Figures 2 and 3).

We conducted a sensitivity analysis to examine the effect of each male-reporting study on the pooled male prevalence. We were unable to find any significant effect on the male pooled prevalence, even after systematically removing studies that had the most influence on the overall pool. For example, among 18 articles surveying male subjects, we found that the pooled prevalence did not significantly change even when we removed the six most influential studies^18,25,34,35,37,44^ in the heterogeneity test and obtained a low heterogeneity (I^2^=38%). The change was only a 1% increase in the overall male pooled prevalence: 27% (95% CI: 25–29%), compared to 26% (95% CI: 24–29%) (Supplementary Figure 2).

We performed a sensitivity analysis on the prevalence of smoking among female students in 17 studies and found no significant change in heterogeneity among these studies. After we systematically removed the eight most influential studies^20,22,23,28,32,34,36,45^, we found a high homogeneity (I^2^=34%), but the pooled prevalence did not significantly differ from the previous calculation. The change was a 1% decrease in the overall female prevalence: 4% (95% CI: 3–5%), compared to 5% (95% CI: 3–7%) (Supplementary Figure 3).

## DISCUSSION

To our knowledge, this study was the first comprehensive meta-analysis performed that aimed to systematically review eligible articles reporting the prevalence of smoking tobacco among higher-education students in the KSA. This study also provided a close look at the current tobacco smoking problem among Saudi college students, compared to national level prevalence and the prevalence in neighboring countries. The results of this study indicate that the pooled estimate of tobacco smoking among college students in the KSA was 17%, which was 5% higher than the average prevalence reported among Saudi daily current smokers aged 15 to 25 years^7^. This indicates that Saudi college students smoke at a higher rate compared to a slightly similar age-group in the nationally representative study. Furthermore, two regional cross-sectional studies showed that the prevalence of smoking reached 12.4% in Yemen and 15.1% in the United Arab Emirates (UAE)^46,47^. To compare these prevalence rates to the findings of the present study, university students in the KSA recorded approximately 5% and 2% higher prevalence of smoking than students in Yemen and the UAE^46,47^, respectively. Based on a similar meta-analysis study, smoking prevalence among college students in the KSA was higher than that found in other countries in the same region, such as Iran, with prevalence of 17% compared to 11.6%, repectively^48^. Overall, Saudi college students in this study had a higher rate of smoking tobacco compared to Saudi current and daily current smokers aged 15 to 25 years and compared to studies conducted in regional countries.

The pooled smoking prevalence among male university students reported in this meta-analysis was 4.5% higher than the national prevalence among Saudi males aged 15 years and older^7^. A meta-analysis study found that Iranian male college students had a smoking prevalence of 19.8%, which is 6.2% lower than what is reported in this meta-analysis for Saudi males^48^. The current study, moreover, did parallel with a nationally representative study that found that Saudi male individuals had a statistical increase in smoking prevalence from 1980 to 2012, compared with 186 countries^3^. The findings of the current study assert that there is a huge difference among the tobacco smoking prevalence rates between male and female college students in the KSA.

In the findings of this meta-analysis, Saudi male college students reported a smoking prevalence that was 21% higher than that of Saudi female college students. This notable difference may be attributed to a limited access to female participants in the KSA. One study reported that the researcher was not able to conduct his research on females because it was culturally unacceptable for a male investigator to survey female students^44^. Another issue of female participation was social desirability bias tied to smoking behavior. Such behavior, especially among women in the KSA, is viewed as destructive to Saudi community values. Therefore, female smokers may be deterred from accurately reporting their smoking status, for fear of societal rejection^49^.

Through examining Saudi female college students’ prevalence of smoking, we found one group of studies that had a prevalence range of 8% to 16%^20,22,28,33,34,36,45^. This unusual range compared to 0–4% may result from the selection at a particular college, making it an exclusive population. For instance, the target populations in most of these studies reporting this range were selected from college students in health-science disciplines^22,33,34,36,45^. Having a satisfactory sample size to conduct the research does not mean it is representative of the whole university population^50^.

In comparison with the 2013 Saudi national survey, this meta-analysis revealed that the number of female college smokers was 4% higher than overall for women aged 15 years or older^7^. Similarly, this meta-analysis showed that the 5% prevalence of Saudi female college smokers was relatively higher than a similar meta-analysis study that reported a 2.2% rate of smoking among Iranian female college students^48^. In contrast, female college students in Yemen had a prevalence of 13%^46^, which was similar to that of Saudi female students (8–16%), but was far from the pooled female prevalence of 5% reported in our study findings.

The majority of studies reported high prevalence when the study population was small and specific, whereas the prevalence would be more representative when the population size is large and diverse. For instance, more than half of the included studies addressed smoking tobacco among health-science-related students; this was not representative of the whole university population, and thus, most of them showed the highest prevalence of smoking. One explanation could be that the majority of health-science-related researchers preferred to conduct their research on convenient and approachable health-science-related students. This technique of sampling could create a potential bias of self-selection, where a student may be unduly influenced by motivation, interest, or health consciousness about the phenomenon^50^.

### Limitations

There were some limitations in the current study. Because of the high variation in instruments, data collection, and study locations among included studies, the result of this meta-analysis could not represent the smoking prevalence of higher-education students in the KSA. However, this was an attempt to estimate and understand the pooled estimate of smoking tobacco prevalence among included studies in this meta-analysis. All of the included studies were cross-sectional in nature, which provided an epidemiological measurement of a certain population of interest rather than examining any association or causation. As was noted, culture barriers play a crucial role in reporting the real prevalence. Thus, this study may have been influenced by the cultural and societal biases reported by some studies, which may have underestimated the actual pooled estimate among Saudi female participants.

## CONCLUSIONS

Tobacco smoking is a public health problem among college students in the Kingdom of Saudi Arabia (KSA). The debate over the prevalence of tobacco smoking has been well investigated. College students in the KSA have a high tobacco smoking prevalence, compared with the national Saudi smoking prevalence and that of neighboring countries. Future studies should use available resources to shift from repeatedly addressing the prevalence of smoking behaviors among college students in the KSA to focusing on intervention and prevention strategies. One idea to monitor the prevalence of smoking is through establishing a tobacco surveillance system that tracks and records Saudi college students’ smoking behaviors. Future research should focus on the psychosocial and economic determinants, from theoretical and experimental designs, as a means of finding strategies that encourage smoking cessation and prevention among college students in the KSA.

## CONFLICTS OF INTEREST

Authors have completed and submitted the ICMJE Form for Disclosure of Potential Conflicts of Interest and none was reported.

## References

[1] Drope J, Schluger N, Cahn Z (2018). The Tobacco Atlas.

[2] Centers for Disease Control and Prevention Global tobacco control.

[3] Ng M, Freeman MK, Fleming TD (2014). Smoking prevalence and cigarette consumption in 187 countries, 1980-2012. JAMA.

[4] Taha S KSA imports tobacco products worth SR13 billion in 4 years. Arab News.

[5] AlBedah AM, Khalil MK (2014). The economic costs of tobacco consumption in the Kingdom of Saudi Arabia. Tob Control.

[6] Alrabah M, Gamaleddin I, Allohidan F (2018). International approaches to tobacco-use cessation programs and policy for adolescents and young adults in Saudi Arabia. Curr Addict Rep.

[7] Moradi-Lakeh M, El Bcheraoui C, Tuffaha M (2015). Tobacco consumption in the Kingdom of Saudi Arabia, 2013: findings from a national survey. BMC Public Health.

[8] Almutairi KM (2014). Smoking among Saudi students: a review of risk factors and early intentions of smoking. J Community Health.

[9] Ameri Z, Mirzakhani F, Nabipour AR, Khanjani N, Sullman MJ (2017). The relationship between religion and risky behaviors among Iranian university students. J Relig Health.

[10] Bader P, Boisclair D, Ferrence R (2011). Effects of tobacco taxation and pricing on smoking behavior in high risk populations: a knowledge synthesis. Int J Environ Res Public Health.

[11] Moher D, Liberati A, Tetzlaff J, Altman FG (2010). Preferred repotting items for systematic review and meta-analyses: the PRISMA statement. Int J Surg.

[12] Russell CK, Gregory DM (2003). Evaluation of qualitative research studies. Evid Based Nurs.

[13] Barendregt JJ, Doi SA, Lee YY, Norman RE, Vos T (2013). Meta-analysis of prevalence. J Epidemiol Community Health.

[14] Doi SA, Barendregt JJ, Khan S, Thalib L, Williams GM (2015). Advances in the meta-analysis of heterogeneous clinical trials I: The inverse variance heterogeneity model. Contemp Clin Trials.

[15] Higgins JPT, Thompson SG, Deeks JJ, Altman DG (2003). Measuring inconsistency in meta-analyses. BMJ.

[16] Tukey JW (1977). Exploratory Data Analysis.

[17] Abdulghani HM, Alrowais NA, Alhaqwi AI (2013). Cigarette smoking among female students in five medical and nonmedical colleges. Int J Gen Med.

[18] Al-Mohaithef M, Chandramohan S (2018). Prevalence of smoking and its associated factors among male college students in Abha, Kingdom of Saudi Arabia: A cross-sectional study. Int J Med Res Health Sci.

[19] Abd El Kader SM, Al Ghamdi AA (2018). Smoking prevalence, attitude, knowledge and practice among applied medical sciences Saudi students in King Abdalaziz university. Int J Pul & Res Sci.

[20] Dar-Odeh NS, Aleithan FA, Alnazzawi AA, Al-Shayyab MH, Abu-Hammad SO, Abu-Hammad OA (2017). Factors affecting oral health determinants in female university students: a cross-sectional survey in Saudi Arabia. Int J Adolesc Med Health.

[21] Azhar A, Alsayed N (2012). Prevalence of smoking among female medical students in Saudi Arabia. Asian Pac J Cancer Prev.

[22] AL-Saegh SZ, Bakarman M, Habadi MI (2017). Prevalence of smoking and its associated factors among female medical students in King Abdulaziz University, KSA, Jeddah. IJIR.

[23] Ansari K, Farooqi FA (2017). Comparison and prevalence of smoking among Saudi females from different departments of the college of applied medical sciences in Dammam. Int Jo Health Sci.

[24] Awan KH, Alrshedan A, Al Kahtani M, Patil S (2016). Waterpipe smoking among health sciences university students: Knowledge, attitude and patterns of use. Saudi Dent J.

[25] Al-Ghaneem SG, Al-Nefisah OS (2016). The prevalence of smoking among male students of Majmaah University, KSA. J Taibah Univ Med Sci.

[26] Ansari T, Alghamdi T, Alzahrani M (2016). Risky health behaviors among students in Majmaah University, Kingdom of Saudi Arabia. J Family Community Med.

[27] Awan KH (2016). Experimentation and correlates of electronic nicotine delivery system (electronic cigarettes) among university students – A cross sectional study. Saudi Dent J.

[28] Koura MR, Al-Dossary AF, Bahnassy AA (2011). Smoking pattern among female college students in Dammam, Saudi Arabia. J Family Community Med.

[29] Mandil A, BinSaeed A, Ahmad S, Al-Dabbagh R, Alsaadi M, Khan M (2010). Smoking among university students: a gender analysis. J Infect Public Health.

[30] Al-Kaabba AF, Saeed AA, Abdalla AM, Hassan HA, Mustafa AA (2011). Prevalence and associated factors of cigarette smoking among medical students at King Fahad Medical City in Riyadh of Saudi Arabia. J Family Community Med.

[31] Allohidan F, Alanazi AK, Azzahrani MK, Alrashoud MR (2017). Knowledge, practice, and attitudes regarding hookah (water pipe) smoking among college students studying health sciences in Riyadh, Saudi Arabia. International Journal of Academic Scientific Research.

[32] AlQahtani JM (2017). Knowledge, attitude and practice of tobacco smoking among health colleges’ students at Najran University, Saudi Arabia: A cross-sectional descriptive study. J Health Spec.

[33] el-Fetoh NMA, Mohammed NA, Alanazi AM, Alruwaily HT, Alenezi OT, Alhowaish JA (2016). Smoking in medical students of Northern Border University, Kingdom of Saudi Arabia. Journal of American Science.

[34] Mansour A, Bakhsh Z (2015). Measuring willingness to accept second-hand smoke exposure. Am J Health Behav.

[35] Shah AH, ElHaddad SA (2015). Oral hygiene behavior, smoking, and perceived oral health problems among university students. J Int Soc Prev Community Dent.

[36] Wali SO (2011). Smoking habits among medical students in Western Saudi Arabia. Saudi Med J.

[37] Mahfouz MS, Alsanosy RM, Gaffar AM, Makeen A (2014). Tobacco use among university students of Jazan Region: gender differences and associated factors. BioMed Res Int.

[38] AlSwuailem AS, AlShehri MK, Al-Sadhan A (2014). Smoking among dental students at King Saud University: Consumption patterns and risk factors. Saudi Dent J.

[39] Al-Haqwi AI, Tamim H, Asery A (2010). Knowledge, attitude and practice of tobacco smoking by medical students in Riyadh, Saudi Arabia. Ann Thorac Med.

[40] Hassan HM, Mahmoud SS, Katasha MK (2014). Tobacco smoking among students of Al-Ghad college for applied medical sciences for male in Riyadh, Saudi Arabia. Int J Med Sci Public Health.

[41] Almogbel YS, Abughosh SM, Almeman AA (2016). Factors associated with the willingness to quit smoking among a cohort of university students in the KSA. J Taibah Univ Med Sci.

[42] Taha AZ, Sabra AA, Al-Mustafa ZZ, Al-Awami HR, Al-Khalaf MA, Al-Momen MM (2010). Water pipe (shisha) smoking among male students of medical colleges in the eastern region of Saudi Arabia. Ann Saudi Med.

[43] Al-Mohamed HI, Amin TT (2010). Pattern and prevalence of smoking among students at King Faisal University, Al Hassa, Saudi Arabia. East Mediterr Health J.

[44] Almutairi KM (2016). Predicting relationship of smoking behavior among male Saudi Arabian college students related to their religious practice. J Relig Health.

[45] Torchyan AA, BinSaeed AA, Aleid YS (2016). Interaction effects of happiness and physical activity on smoking initiation. Am J Health Behav.

[46] Nasser AM, Salah BA, Regassa LT, Alhakimy AAS, Zhang X (2018). Smoking prevalence, attitudes and associated factors among students in health-related departments of community college in rural Yemen. Tob Induc Dis.

[47] Mandil A, Hussein A, Omer H, Turki G, Gaber I (2007). Characteristics and risk factors of tobacco consumption among University of Sharjah students, 2005. East Mediterr Health J.

[48] Haghdoost AA, Moosazadeh M (2013). The prevalence of cigarette smoking among students of Iran’s universities: A systematic review and meta-analysis. J Res Med Sci.

[49] Ghouri N, Atcha M, Sheikh A (2006). Influence of Islam on smoking among Muslims. BMJ.

[50] Portney LG, Mary PW (2015). Foundations of Clinical Research: Applications to Practice.

