# Supplementary material for: Smoking tobacco prevalence among college students in the Kingdom of Saudi Arabia: Systematic review and meta-analysis
Source: Tob Induc Dis. 2019 Apr 19;17:35. doi: 10.18332/tid/105843 (PMC6662783; doi:10.18332/tid/105843)
Supplement: Supplementary file 1 [file TID-17-35-s1.pdf]

Supplementary Table 1. Search strategy

Databases	Search Keywords	Total
**PubMed (2010-2018)**	(((Smoking[Title/Abstract] OR Tobacco[Title/Abstract] OR Cigarette[Title/Abstract] OR Waterpipe[Title/Abstract])) AND (College[Title/Abstract] OR University[Title/Abstract] OR Students[Title/Abstract])) AND (Saudi[Title/Abstract] OR KSA[Title/Abstract])	**120**
**Science Direct (2010-2018)**	(Smoking OR Tobacco OR Cigarette OR Waterpipe) AND (College OR University OR Students) AND (Saudi OR KSA); **Note: Find articles in: Title, Abstract, Keywords. Restrict research to: Review articles & Research articles.**	**16**
**APA PsycNET (2010-2018)**	Any Field: smoking OR Any Field: Tobacco OR Any Field: Cigarette OR Any Field: Waterpipe AND Any Field: College OR Any Field: University OR Any Field: Students AND Any Field: Saudi OR Any Field: KSA AND Peer-Reviewed Journals only AND Year: 2000 To 2018	**71**
**Web of Science (2010-2018)**	TITLE: (smoking OR tobacco OR Cigarette OR Waterpipe) AND TITLE: (college OR University OR Students) AND TOPIC:(Saudi OR KSA)	**46**
**CINAHL (2010-2018)**	AB (smoking OR tobacco OR cigarette Or Waterpipe) AND AB (College Or University Or Students) AND AB (Saudi OR KSA)	**35**

Supplementary Table 2. Quality assessment of included articles based on Russell and Gregory's guidelines[12].

#	Source	Year	Q1	Q2	Q3	Q4	Q5	Quality Score
1	Abdulghani,[17]	2017	Yes	Yes	No	Yes	Yes	4
2	Al-Mohaithef,[18]	2018	Yes	Yes	Yes	No	Yes	4
3	Abd El Kader,[19]	2018	Yes	Yes	No	Yes	Yes	4
4	Dar-Odeh,[20]	2017	Yes	Yes	No	Yes	Yes	4
5	Azhar,[21]	2012	Yes	Yes	Yes	Yes	Yes	5
6	AL-Saegh,[22]	2017	Yes	Yes	Yes	No	Yes	4
7	Ansari,[23]	2017	Yes	Yes	Yes	No	Yes	4
8	Awan,[24]	2016	Yes	Yes	No	Yes	Yes	4
9	Al-Ghaneem,[25]	2016	Yes	Yes	Yes	Yes	Yes	5
10	Ansari,[26]	2016	Yes	Yes	No	Yes	Yes	4
11	Awan,[27]	2016	Yes	Yes	No	Yes	Yes	4
12	Koura,[28]	2011	Yes	Yes	Yes	No	Yes	4
13	Mandil,[29]	2010	Yes	Yes	Yes	Yes	Yes	5
14	Al-Kaabba,[30]	2011	Yes	Yes	No	Yes	Yes	4
15	Allohidan,[31]	2017	Yes	Yes	Yes	No	Yes	4
16	AlQahtani,[32]	2017	Yes	Yes	No	Yes	Yes	4
17	el-Fetoh,[33]	2016	Yes	Yes	No	Yes	Yes	4
18	Mansour,[34]	2015	Yes	Yes	No	Yes	Yes	4
19	Shah,[35]	2015	Yes	Yes	Yes	No	Yes	4
20	Wali,[36]	2011	Yes	Yes	No	Yes	Yes	4
21	Mahfouz,[37]	2014	Yes	Yes	Yes	Yes	Yes	5
22	AlSwuailem,[38]	2014	Yes	Yes	No	Yes	Yes	4
23	Al-Haqwi,[39]	2010	Yes	Yes	No	Yes	Yes	4
24	Hassan,[40]	2014	Yes	Yes	No	Yes	Yes	4
25	Almogbel,[41]	2016	Yes	Yes	No	Yes	Yes	4
26	Taha,[42]	2010	Yes	Yes	Yes	Yes	Yes	5
27	Al-Mohamed,[43]	2010	Yes	Yes	Yes	No	Yes	4
28	Almutairi,[44]	2016	Yes	Yes	No	Yes	Yes	4
29	Torchyan,[45]	2016	Yes	Yes	Yes	No	Yes	4

© 2019 Alotaibi SA.
