# Supplementary material for: Smoking tobacco prevalence among college students in the Kingdom of Saudi Arabia: Systematic review and meta-analysis
Source: Tob Induc Dis. 2019 Apr 19;17:35. doi: 10.18332/tid/105843 (PMC6662783; doi:10.18332/tid/105843)
Supplement: Supplementary file 2 [file TID-17-35-s2.pdf]

Supplementary Figure 1. Pooled estimate of smoking tobacco among all included studies (n=29).

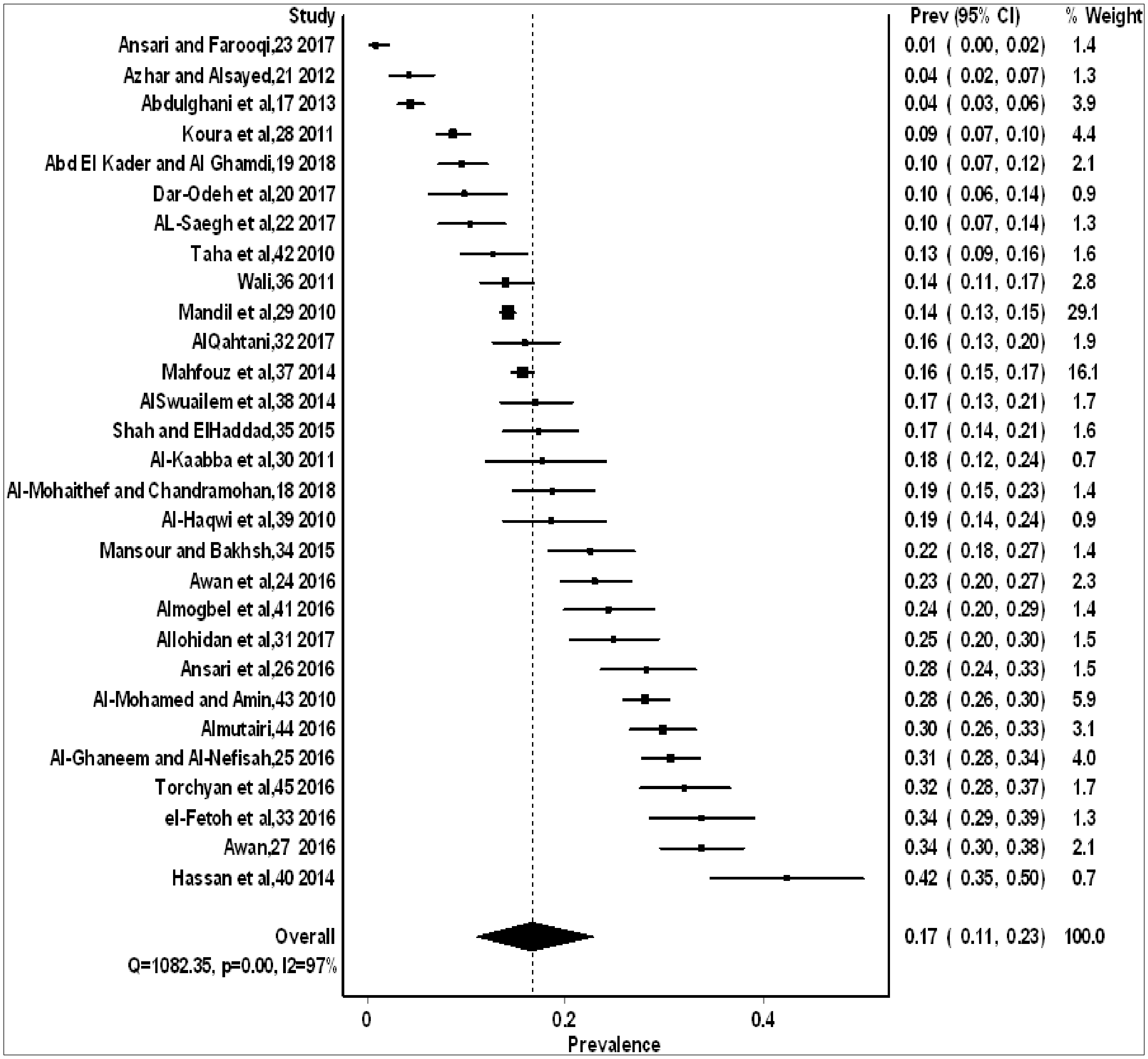

Supplementary Figure 2. Male prevalence of smoking after conducting the sensitivity analysis.

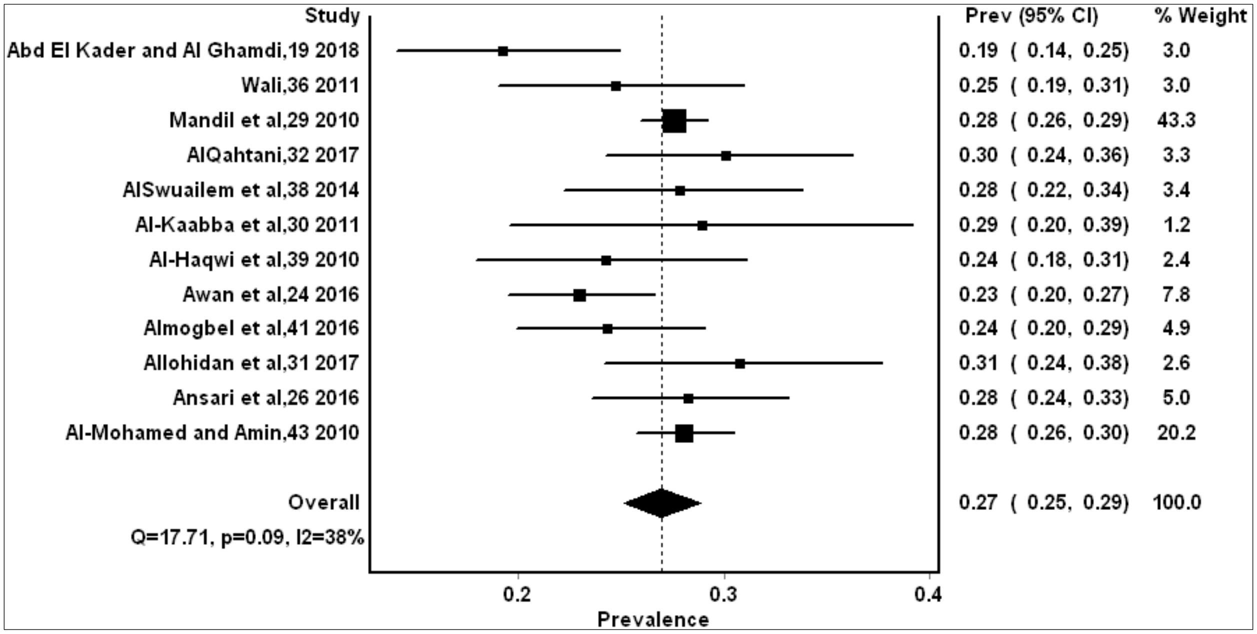

Supplementary Figure 3. Female prevalence of smoking after conducting the sensitivity analysis.

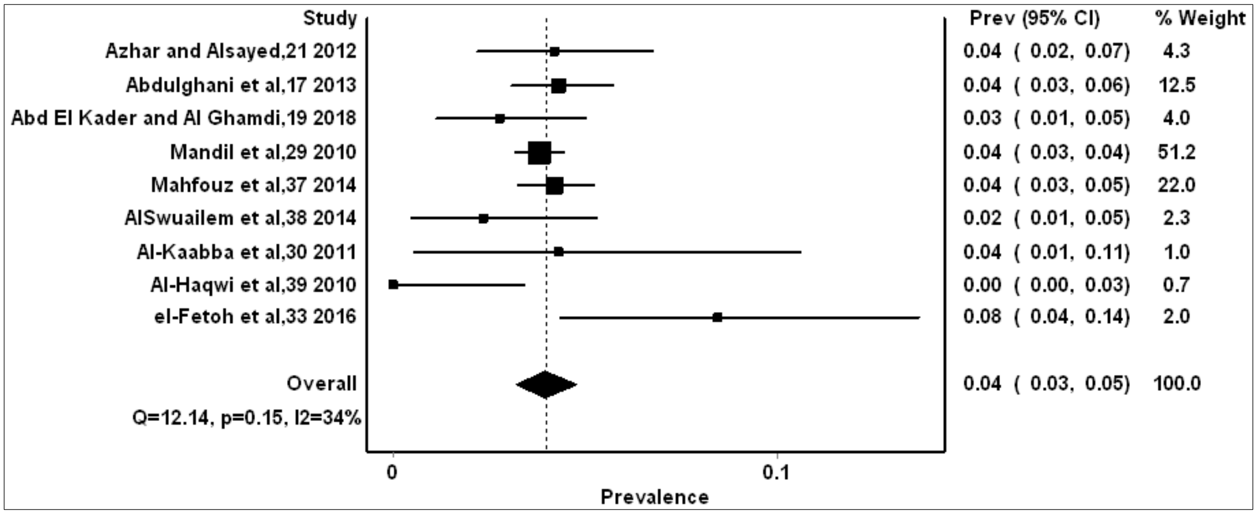

© 2019 Alotaibi SA.
